# Collaborative care for patients with bipolar disorder: a randomised controlled trial

**DOI:** 10.1186/1471-244X-11-133

**Published:** 2011-08-17

**Authors:** Trijntje YG van der Voort, Berno van Meijel, Peter JJ Goossens, Janwillem Renes, Aartjan TF Beekman, Ralph W Kupka

**Affiliations:** 1 GGZ ingeest/VU University Medical Center, dept. of Psychiatry, Amsterdam, the Netherlands; Inholland University of Applied Sciences, Research Group Mental Health Nursing, Amsterdam, the Netherlands; Dimence Mental Health, Deventer, the Netherlands; 2 Inholland University of Applied Sciences, Research Group Mental Health Nursing, Amsterdam, the Netherlands; 3 Dimence Mental Health, Deventer, the Netherlands; Radboud University Medical Center, Nijmegen, the Netherlands; Saxion University of Applied Sciences, Deventer, the Netherlands; 4 Altrecht Institute for Mental Health Care, Utrecht, the Netherlands; 5 VU University Medical Center, dept. of Psychiatry, Amsterdam, the Netherlands; 6 VU University Medical Center, dept. of Psychiatry, Amsterdam, the Netherlands; Altrecht Institute for Mental Health Care, Utrecht, The Netherlands

## Abstract

**Background:**

Bipolar disorder is a severe mental illness with serious consequences for daily living of patients and their caregivers. Care as usual primarily consists of pharmacotherapy and supportive treatment. However, a substantial number of patients show a suboptimal response to treatment and still suffer from frequent episodes, persistent interepisodic symptoms and poor social functioning. Both psychiatric and somatic comorbid disorders are frequent, especially personality disorders, substance abuse, cardiovascular diseases and diabetes. Multidisciplinary collaboration of professionals is needed to combine all expertise in order to achieve high-quality integrated treatment. 'Collaborative Care' is a treatment method that could meet these needs. Several studies have shown promising effects of these integrated treatment programs for patients with bipolar disorder. In this article we describe a research protocol concerning a study on the effects of Collaborative Care for patients with bipolar disorder in the Netherlands.

**Methods/design:**

The study concerns a two-armed cluster randomised clinical trial to evaluate the effectiveness of Collaborative Care (CC) in comparison with Care as usual (CAU) in outpatient clinics for bipolar disorder or mood disorders in general. Collaborative Care includes individually tailored interventions, aimed at personal goals set by the patient. The patient, his caregiver, the nurse and the psychiatrist all are part of the Collaborative Care team. Elements of the program are: contracting and shared decision making; psycho education; problem solving treatment; systematic relapse prevention; monitoring of outcomes and pharmacotherapy. Nurses coordinate the program. Nurses and psychiatrists in the intervention group will be trained in the intervention. The effects will be measured at baseline, 6 months and 12 months. Primary outcomes are psychosocial functioning, psychiatric symptoms, and quality of life. Caregiver outcomes are burden and satisfaction with care.

**Discussion:**

Several ways to enhance the quality of this study are described, as well as some limitations caused by the complexities of naturalistic treatment settings where not all influencing factors on an intervention and the outcomes can be controlled.

**Trial Registration:**

The Netherlands Trial Registry, NTR2600.

## Background

Bipolar disorder is a severe mental illness with an estimated lifetime prevalence of 1.5 to 2%[1]. It is characterized by recurrent episodes of extreme mood changes. Several studies [2-4] show that on average patients suffer from manic, depressive, hypomanic or mixed symptoms for about half of the time despite treatment. Even during so-called euthymic periods, i.e. when they are formally not in a mood episode, many patients suffer from subsyndromal symptoms that negatively influence their quality of life [5]. Informal caregivers also suffer a substantial burden by the illness, not only during episodes [6,7].

Treatment of bipolar disorders in the Netherlands primarily consists of pharmacotherapy, supportive treatment, and psycho education, sometimes combined with improvement of self-management skills or psychotherapy. Many patients respond well to this treatment and may stabilize for longer periods. However, a substantial number of patients do not respond adequately to these treatment efforts. They suffer from frequent episodes, persisting symptoms, cognitive problems, limited social support and poor social functioning. Comorbid psychiatric disorders, such as personality disorders and substance abuse, are common, as are somatic disorders i.e. cardiovascular disease, partly associated with prolonged use of maintenance medication [8,9].

Multidisciplinary collaboration of professionals is needed to involve optimal specialist skills on all aspects of the disorder, and to properly combine all expertise in order to achieve an optimally integrated care [10]. The Dutch guideline for the treatment of bipolar disorders [11] recommends such an integrated treatment. The guideline advises that treatment be targeted at reducing symptoms, acceptance of the illness, promoting treatment adherence, stabilising social rhythm, recognising early signs and symptoms and take proper actions when these occur, diminishing social problems and maximising social participation. It is advised to actively engage caregivers or family members in this treatment. Although it is widely acknowledged that integrated treatment programs can be of great value for patients with bipolar disorder, research on the effectiveness of such programs for patients with a bipolar disorder is scarce.

Collaborative Care (CC) programs have been developed to integrate multidisciplinary care for complex disorders and to date, three research projects show promising results with bipolar patients. Bauer et al. [12,13] have implemented a CC model for patients with bipolar disorder and measured the effects in two randomised controlled trials. Patients participated in the Life Goals Program. The intervention consisted of improving patients' self-management skills through psycho education; supporting providers' decision making through simplified practice guidelines; and enhancing access to care and continuity of care. The care manager, a nurse or a social worker, provided actively outreaching care when a patient was at risk of losing contact with mental health workers. Each patient made a 'relapse prevention plan', aimed at the early recognition of relapses. The care manger contacted the patient at least every three months. The program also provides guidelines for specific pharmacotherapy. Bauer et al. [12,13] found significantly reduced number of weeks in manic episodes in the experimental group, although no significant effect was found on weeks depressed. Patients showed improved social functioning, quality of life and treatment satisfaction. Total costs were similar in both groups. The Life Goals Program was extended by Kilbourne et al. [14,15], to address the many medical comorbidities present in patients with bipolar disorders.

Simon et al. [16,17] have explored the effects of adding intensive nursing care to treatment of patients with bipolar disorder. This consisted of an emergency plan, monthly telephone calls in which symptoms were monitored, sharing of information between nurse and psychiatrist, and outreach interventions such as crisis intervention. Patients were given the choice of participation in the Life Goals Program, mentioned above. After receiving the intervention over a period of one or two years, patients in the experimental group showed significant less severe manic symptoms of shorter duration. Also in this study, there was no effect on depressive symptoms. Extra costs of this treatment were relatively low compared to the control group.

Suppes et al. [18] studied the effects of the Texas Medication Algorithm project in patients with bipolar disorder. This project aimed to develop and implement algorithms for pharmacotherapy for bipolar patients. Clinics implementing this guideline were compared in a RCT to clinics which did not use the guideline. In the experimental group, a coordinator was added to the care team. This coordinator provided psycho education to the patient and the family. He kept in contact with the patient, and assessed symptoms and side effects of medication prior to each consultation with the psychiatrist. The coordinator informed the psychiatrist about the results of these assessments. The aim was to achieve a higher efficiency of the psychiatric consultations. The results of the study show a significant improvement of psychiatric symptoms of patients in the intervention group in the first three months. During the next nine months, patients in the control group improved as well, but less than patients in the intervention group. Patients in the intervention group reported significant improvement of manic, but not depressive and psychotic symptoms.

It is striking that all three studies show improvement of manic but not depressive symptoms.

In the Netherlands, until now no studies have been performed on the effects of CC for patients with bipolar disorder. The current study will be the first to implement CC for patients with bipolar disorder, and moreover, to add a specific intervention to improve depression. Furthermore, CC appears to offer good possibilities to strengthen the position of the nurse in the care process for patients with a bipolar disorder, both at the organizational and content levels.

In this article we describe the study protocol for investigating the effectiveness of a CC program for patients with bipolar disorder. We set out the following three research questions to answer:

A What are the effects of a CC program, compared to Care as Usual (CAU), for patients with bipolar disorder, with regard to their psychosocial functioning, psychiatric symptoms, quality of life, attitudes towards medication, mastery, and satisfaction with care?

B What are the effects of a CC program, compared to CAU, for informal caregivers of patients with a bipolar disorder, with regard to the experienced burden and satisfaction with care?

C What is the cost effectiveness of a CC Program compared to CAU?

## Methods/design

### Design

A pragmatic two-armed cluster randomized controlled trial (RCT) will be carried out in mental health outpatient clinics in the Netherlands. A Collaborative Care program will be executed in the experimental condition, whereas the control condition will continue to deliver Care as Usual (CAU). CC will be performed by the CC-team, with mental health nurses in a coordinating role. Baseline measurements will be obtained at inclusion and follow-up measurements will take place at 6 months and 12 months. The primary outcome measures are psychosocial functioning, symptoms, and quality of life. Secondary outcome measures are mastery, attitude towards medication and satisfaction with care. Family members or friends of patients will be asked to fill in questionnaires regarding perceived burden and satisfaction with care.

### Study on Care as Usual

The level of CAU may vary considerably among the various outpatients clinics in the Netherlands, and there is a lack of clarity about the specific content of CAU for patients with bipolar disorder. However, there is more detailed knowledge available about care provided in some highly specialized centres for patients with bipolar disorder that may resemble CC. In the Netherlands, about 20.000 patients receive care for bipolar disorder but it is estimated that only 1500 patients receive this care in these specialized centres. Hence, about 18.500 patients receive CAU at a less specific level. Our presumption is that CC will differ considerably from CAU, which we expect to be mainly supportive and not systematically delivered. In order to increase our knowledge about the characteristics of CAU another study has started to describe current CAU for bipolar disorders in the Netherlands. In this study, psychiatrists and patients are being asked to describe the care actually delivered to patients with bipolar disorder, by means of questionnaires. These data will be used in the current study: (1) to objectively describe CAU; (2) to select sites (based on our inclusion criteria) that can be included in our CC-trial; and (3) to perform the matching of sites for the (cluster) randomization. Furthermore, if outpatient care teams are interested in participating in the CC-trial, we will interview two members of that team to investigate the nature and intensity of care currently delivered to patients with bipolar disorder. Clinics that currently have a level of CAU that does not already resemble CC can be considered for randomisation.

### Randomisation

In this trial we will use clustered randomisation with outpatient clinic teams as clusters. Randomisation will be performed as follows: the CAU-study as well as the interviews with team members provide us with insight in the nature and intensity of CAU at that site. This enables us to describe CAU more precisely and formulate criteria for inclusion. Sites that meet the criteria for CAU will be invited to join in the CC-trial. Teams will be matched for characteristics of care offered to bipolar patients, as well as organisational characteristics such as team composition or type of organisation. One team of every pair will be randomly assigned to the experimental condition, and the other to the control condition. Patients in both teams who meet the inclusion criteria will be asked to participate in the study.

### Respondents

Patients who meet the following inclusion criteria will be included:

• diagnosis of bipolar disorder according to DSM- IV-TR [19];

• age 18-65 years.

Exclusion criteria:

• Acute phase of severe depression or mania (CGI-BP: degree of illness score 6 or score 7).

• A very stable course of illness (during the last year) that allows low intensity of treatment (2-4 consults with the psychiatrist per year suffice), to be judged by the treating psychiatrist.

• Insufficient command of the Dutch language.

• Not able or willing to give informed consent.

If the patient consents, a family member or friend will also be asked to participate in the study, according to the following inclusion criteria:

• The family member or friend is assigned by the patient.

• The family member or friend is 18 years or older.

• The family member or friend has a sufficient command of the Dutch language.

• The family member or friend is able and willing to give informed consent.

### Blinding

Since the current study is a pragmatic intervention study, blinding of patients and professionals for care modality is not possible. However, blinding is performed in the procedure of randomisation and the statistical analysis. Furthermore, the research assistant who performs interviews by telephone to administer the Life Chart, will be blind to the condition of the respondent.

### The intervention

CC will be recovery-oriented, meaning that it supports the personal process of the patient towards redefining and achieving his goals in life despite the disorder. It will consist of the following systematically delivered elements of treatment:

• The formation of a Collaborative Care team, including at least the patient, the nurse and the psychiatrist, were all decisions concerning treatment and care will be made, using shared decision making. If possible, a family member or friend will be engaged in the treatment and be part of the CC team. Teams meet every three months. Coordination of care and continuity of care is provided by the nurse.

• Contracting. Agreement on the most important problems to be worked on, and on which treatment is needed to help the patient achieve his personal goals, will be striven for. A treatment plan will be made, formulated as a contract, in which goals and treatment activities are recorded.

• Working with the treatment plan based on systematic care needs assessment, and that will focus specifically on recovery-oriented goals, defined by the patient. Systematic monitoring of outcomes.

• Psycho education [20,21]), provided as a course directed at a group of patients and caregivers.

• Problem Solving Treatment (PST) [22,23], delivered by the nurse.

• Mood charting by means of the Life Chart Method [24] and recognising early warning signs of relapse [25-27].

• Pharmacotherapy and somatic care, as usual. In addition, structured and continuous monitoring of the effects.

• If indicated, specialised PST will be delivered (1) to patients who remain depressed, aimed at increasing the amount of pleasant or fulfilling activities, and (2) to patients with wishes or care needs on the subject of participation in society, aimed at rehabilitation.

These interventions will be tailored to the individual needs of the patient.

In the CC trial, CC will be implemented in the experimental teams by the use of the Replicating Effective Programs framework (REP) [28]. This framework appears to be well-suited for the implementation of our program as it specifies steps needed to maximise fidelity while allowing opportunities for flexibility. REP consists of four phases: pre-conditions (e.g., identifying need, target population, and suitable intervention), pre-implementation (e.g., intervention packaging), implementation (e.g., package dissemination, training, technical assistance, and evaluation), and maintenance and evolution (e.g., preparing the intervention for sustainability). Key components of REP, including intervention packaging, training, technical assistance, and fidelity assessment are crucial to the implementation of effective interventions in health care.

A manual-based training program will be developed by the researchers with assistance of an expert panel consisting of expert nurses, psychiatrists, patients and family members. Nurses in the experimental condition will receive a three-day training program, and psychiatrists and psychologists will receive parts of this training. The trained teams will perform CC during one year. They will be primarily responsible for the coordination and continuity of treatment and care for patients with bipolar disorder. The primary investigator (TV) will supervise the nurses intensively during the performing stage of the intervention, by weekly contact by telephone and regular meetings in the facility with colleagues who also participate in the study. Fidelity will be assessed by means of checklists filled out by the nurses in the experimental group, and by randomly checking the electronic files of patients, by the primary investigator.

Nurses in the control group will be performing Care As Usual, of which the content will be assessed by the researcher at baseline, by means of the checklist mentioned before. The Care consumption will be measured with the TIC-P questionnaire in both groups.

### Measures (Table 1)

Measurements will take place at baseline and after 6 and 12 months of follow-up in patients, clinicians and family members or friends of the patients. All questionnaires are available in Dutch. At baseline, demographics, illness history and current characteristics of bipolar disorder will be recorded with the Questionnaire for Bipolar Illness (QBP-NL), that has been used previously in a large naturalistic cohort study [29,30]. We will measure overall functioning with the Functioning Assessment Short Test (FAST-NL-P). This questionnaire has been shown to have good psychometric proportions [31]. The current severity of the disorder will be measured with the Clinical Global Impression for Bipolar Disorder (CGI-BP) [32], rating manic and depressive symptoms separately, as well as the overall severity of bipolar disorder. Symptoms will be assessed with the Brief Symptom Inventory (BSI), which is the short version of the SCL-90 [33] and shows good psychometric proportions. Patients will fill in the BSI, which takes about 10 minutes to complete. Depressive symptoms will be measured with the Quick Inventory for Depressive Symptoms (QIDS-SR), a 16-item self report scale with excellent psychometric proportions [11]. Manic symptoms will be assessed with the Altman Self Rating Mania Scale (ASRM), which has shown satisfying psychometric qualities [34]. Course of illness and recurrences of mood episodes will be assessed with the Life Chart Method [35] during a telephone interview by a research assistant [36]. Quality of life will be assessed with the World Health Organisation Quality of Life -short version (WHOQoL-Bref), a widely used instrument with good psychometric proportions [37]. Assessment of needs will be measured by the CANSAS-P [38], of which validity and reliability proved to be satisfying [39,40]. Mastery will be measured with the Sense of Mastery Scale, which is widely used and is found valid and reliable [41]. Satisfaction with care will be measured with a Visual Analogue Scale (VAS), and costs with the TiC-P [42]. Information on Attitude on Drugs will be gathered with the Drugs Attitude Inventory (DAI-10) [43], which has been proved valid. Burden perceived by caregivers will be assessed with the Involvement Evaluation Questionnaire (IEQ), a self report list which is widely used and has been found valid [44]. Caregivers will also be asked to score satisfaction with care on a Visual Analogue Scale (VAS).

**Table 1:** Measures in experimental group as well as in control group

Instrument	Filled in by	T0	T6	T12
QBP-NL	p/c	X		

CGI-BP	C	X	x	X

BSI	P	X	x	X

IDS-SR	P	X	x	X

ASRM	P	X	x	X

LIFE	P	X	x	X

FAST	P	X	x	X

WHOQoL-bref	P	X	x	X

CANSAS	P	X	x	X

Mastery	P	X	x	X

TIC-P	P	X	x	X

DAI-10	P	X	x	X

IEQ	F	X	x	X

VAS	p/f	X	x	X

P = patient; F = family or friend (informal caregiver); C = Clinician or professional caregiver (psychiatrist or psychologist)

### Statistical analysis

Primary outcomes are psychosocial functioning; course, prevalence and severity of psychiatric symptoms and quality of life.

Data will be analyzed according to the intention to treat-principle. Baseline comparability of the experimental and control groups in demographic and clinical variables will be evaluated with ANOVA/independent samples t-test and Chi-square tests. Differences in outcome between CC and CAU will be evaluated by means of (mixed model) analysis of covariance. A group*time-interaction term will be entered into the model to test for a difference in treatment effect over time. In analyzing a specific outcome variable, the baseline score of that variable will be used as covariate. The analysis will be extended using multilevel analysis that takes the nesting of measurements into account. In the multilevel analyses we consider location as the first level, patient as the second level, and the three measurements as a third level, using the principles of Twisk [45]. Finally we will take into account to what extent patients were exposed to the intervention by analysing the dose-effect relationship.

### Power calculation

The power calculation concerns the comparison at T12 compared to T0 between the two groups (CC vs. CAU). We were not able to detect studies sufficiently comparable to ours to find a basis for estimating an effect size. We assume the effect size Cohen's d = 0,5, because we consider this a clinically relevant effect. Setting α < .05 (two-tailed), and the power (1-β) = .80, the required sample size is 2 × 63 patients. In cases of clustered randomisation the standard is to add 25% to this amount (n = 2 × 79). Taking into account an expected loss of 30%, a sample of 103 patients in each group is needed.

### Ethical considerations

This study protocol has been reviewed by the Scientific Committee of the EMGO Institute of VU University Medical Center in Amsterdam, The Netherlands, and by the Research Committee of Inholland University for Applied Sciences, Amsterdam, and has been approved for by the Medical Ethical Committee of the VU University Medical Centre for final approval. Patients will obtain oral and written information about the study and will be asked to sign an informed consent form if they are willing to participate. They will give informed consent for participation of a specific caregiver, who will also be asked to give separate informed consent.

## Discussion

This pragmatic trial will to our knowledge be the first to evaluate the effectiveness of recovery oriented Collaborative Care for patients with bipolar disorder outside the United States. Our study will assess primarily the effects of CC on functioning, psychiatric symptoms and quality of life. We expect that CC contributes to an increased quality of care for these patients.

The quality of the study is enhanced in four ways. Firstly, we included the expertise of patients, informal caregivers, psychiatrists and nurses during the process of development of the CC intervention. Secondly, we carefully planned implementation of CC in the experimental group, with the three days of training, weekly coaching for the nurses, regular meetings with the group of nurses that perform CC, and fidelity assessments. In this way we intend to improve intervention compliance in the experimental group. Thirdly, we apply very few a priori exclusion criteria, hence we will be able to generalise our findings to a large group of outpatients with bipolar disorder. Finally, the interviewing research assistant is blinded for the treatment condition of the respondents, while administering the Life Chart by telephone.

Besides these strong points, this study has some limitations, which should be acknowledged. The main limitation is that patients and professional care providers cannot be blinded for the treatment condition to which they will be randomised. This is a possible source of bias, as patients as well as nurses in the intervention group may be more actively involved in the treatment because they expect it to work, which may enhance the effect of the intervention we find. A second limitation is the possibility that participation in the study and being exposed to follow-up assessments may affect the outcome of the CAU control group. This may decrease the contrast between the two groups and thus the probability to find statistically significant differences between the CC and the CAU condition. The third limitation is that it is not possible to discern which component of CC contributed to the effect.

Limitations of this kind are inevitable in a pragmatic trial, because of the complexities of naturalistic treatment settings where not all influencing factors on our intervention and the outcomes can be controlled. Despite this, we hypothesize that this program will improve the quality of care for patients with bipolar disorder, thereby optimizing symptomatic recovery, level of functioning and in the end their quality of life.

## Competing interests

The authors declare that they have no competing interests.

## Authors' contributions

TV drafted this paper and it was modified by all other authors. AB, RK, BM, PG, JR en TV contributed to the design of the study protocol. TV and BM designed the intervention protocol, which was discussed and enhanced with the expert panel on two occasions. All authors contributed to and approved the final manuscript.

## Pre-publication history

The pre-publication history for this paper can be accessed here:

http://www.biomedcentral.com/1471-244X/11/133/prepub
